# Supplementary material for: Insights into de-mixing and morphology modulation in coacervate-membrane interactions from integrating experiments and simulations
Source: Commun Chem. 2025 Dec 10;9:7. doi: 10.1038/s42004-025-01810-w (PMC12770480; doi:10.1038/s42004-025-01810-w)
Supplement: Supplementary file 2 — SI [file 42004_2025_1810_MOESM2_ESM.pdf]

Supporting Information for

Insights into de-mixing and morphology modulation in coacervate-membrane interactions from integrating experiments and simulations

Sayantan Mondal,[a§] Agustín Mangiarotti,[b§] Rumiana Dimova,[b*] and Qiang Cui,[acd*]

[a] Department of Chemistry, Boston University, 590 Commonwealth Avenue, Boston, MA 02215, USA.

[b] Max Planck Institute of Colloids and Interfaces, Science Park Golm, 14476 Potsdam, Germany.

[c] Department of Physics, Boston University, 590 Commonwealth Avenue, Boston, MA 02215, USA.

[d] Department of Biomedical Engineering, Boston University, 44 Cummington Mall, Boston, MA 02215, USA.

[§] S.M. and A.M. *contributed equally*

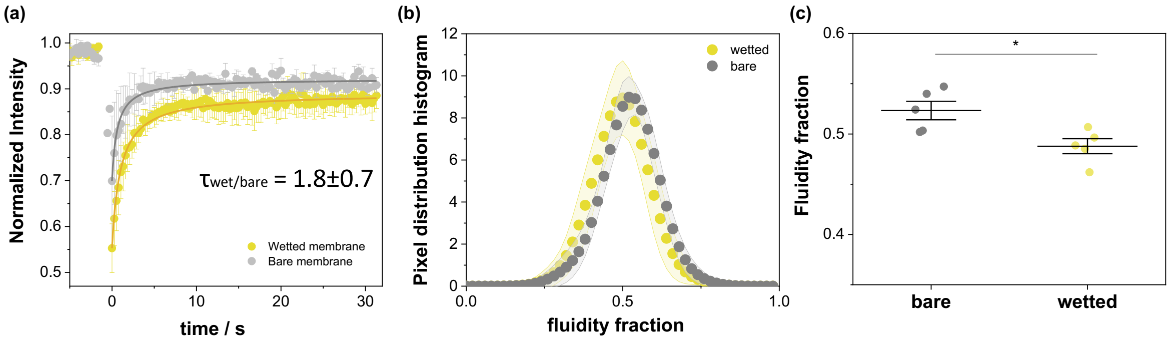

Figure S1: Effects on diffusion coefficient and membrane packing for pure DOPC GUVs in contact with K_{10}/D_{10} coacervates at 15 mM KCl and 0.5mM $MgCl_2$. (a) FRAP of the ATTO 647N-DOPE dye on membrane segments wetted by the coacervate (yellow) and the bare membrane (gray). Data are shown as mean±SD, n=10. (b) Pixel distribution histograms for the membrane wetted and bare segments. The histograms are shown as mean±SD (n=5). (c) Center of mass of the histograms shown in (c). The wetted membrane segments display a reduced fluidity fraction (higher packing) compared to the bare segments. Individual data points are shown as circles and the lines correspond to mean±SD. The differences are significant, p<0.05, ANOVA and Tukey post-test analysis.

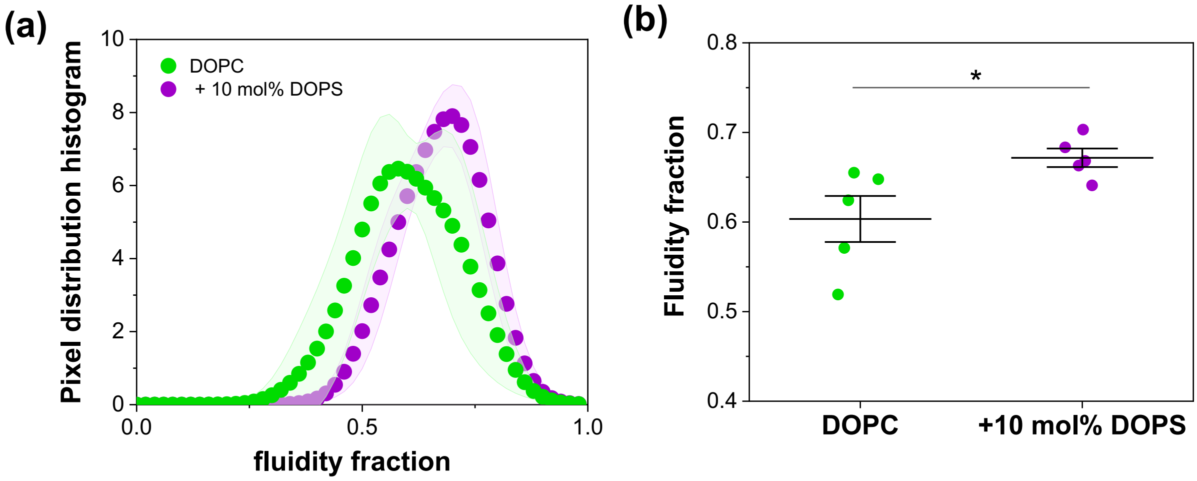

Figure S2: Membrane packing measured for DOPC and DOPC:DOPS 9:1 membranes labeled with 0.5 mol% LAURDAN (in absence of coacervates). (a) Pixel distribution histograms for the DOPC and DOPC:DOPS 9:1 vesicles. The histograms are shown as mean±SD (n=5). (b) Center of mass of the histograms shown in (a). Adding DOPS increases the fluidity fraction (lower packing) compared to the pure DOPC membranes. Individual data points are shown as circles and the lines correspond to mean±SD. The differences are significant, p<0.05, ANOVA and Tukey post-test analysis.

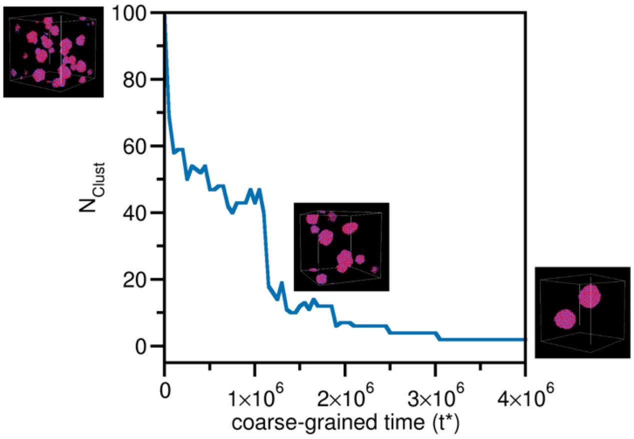

Figure S3. Number of clusters against coarse grained simulation time in the Generic model framework. The plot shows two long lived clusters out of which we took the bigger one (~1200 chains) and used it as a preformed coacervate with the unilamellar Cooke model vesicle. Three representative snapshots of the system are provided to show the formation of larger droplets starting from smaller ones.

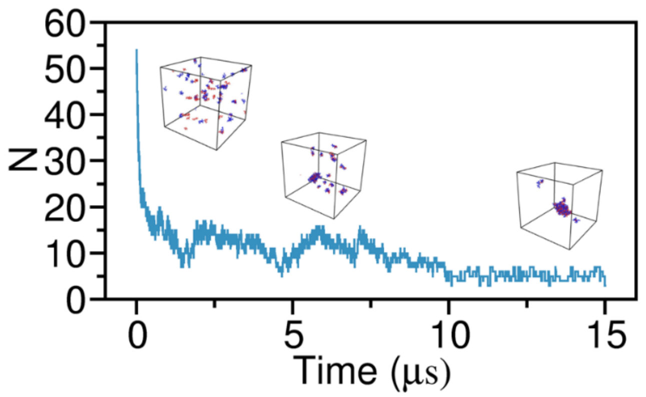

Figure S4. Number of clusters (N) against coarse grained simulation time in the MARTINI model framework. The plot shows four long lived clusters out of which we took the biggest one (~60 chains) and used it as a preformed coacervate with the MARTINI bilayer patches. The other three clusters are actually polyelectrolyte dimers (as can be seen from Figure 1c in the main text). Three representative snapshots of the system are provided to show the formation of larger droplets starting from smaller ones.

Table S1. Modified non-bonded interaction parameters (in kJ/mol unit) between lipid heads and polyelectrolytes. These are calculated as $\boldsymbol{\varepsilon^{LJ}_{mod} = 1.20\ \varepsilon^{LJ}_{original}}$.

Protein \ Lipid	Q1	Q5	SN4a	N4a
P2	5.9172	6.4896	3.7920	4.2240
SC3	3.4344	1.7556	2.4480	2.5300
SQ4p	3.9840	6.9240	7.1628	6.2790
SQ5n	3.9840	7.2240	6.5112	5.9190

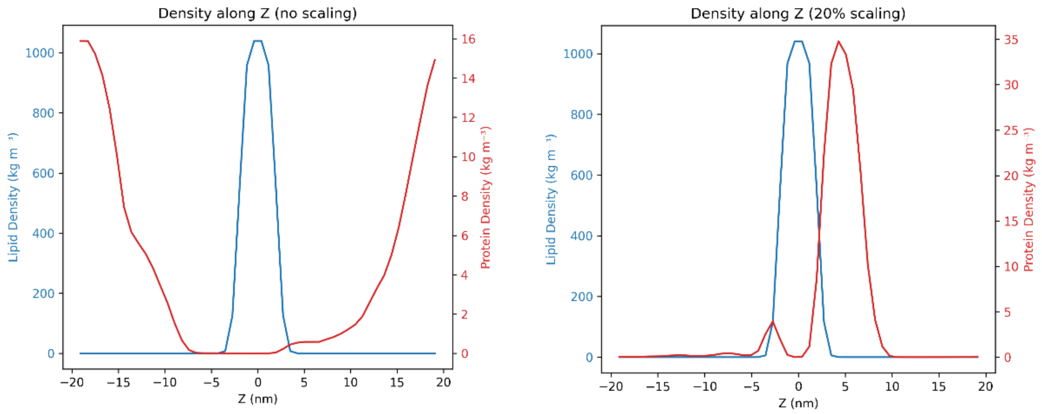

Figure S5. Density profiles of lipids (pure DOPC bilayer) and proteins for the unscaled MARTINI model (left panel) and the rescaled MARTINI model with a 20% increase in protein–lipid interaction strength (right panel). In the unscaled system, the protein remains distant from the bilayer, with an average position around Z = 0 nm. In contrast, the rescaled system shows overlapping density profiles of lipids and proteins, indicating protein adsorption onto the bilayer. The small peak to the left of the lipid density profile in the scaled system corresponds to a few polyelectrolyte molecules from the dilute phase adsorbed onto the opposite leaflet of the bilayer.

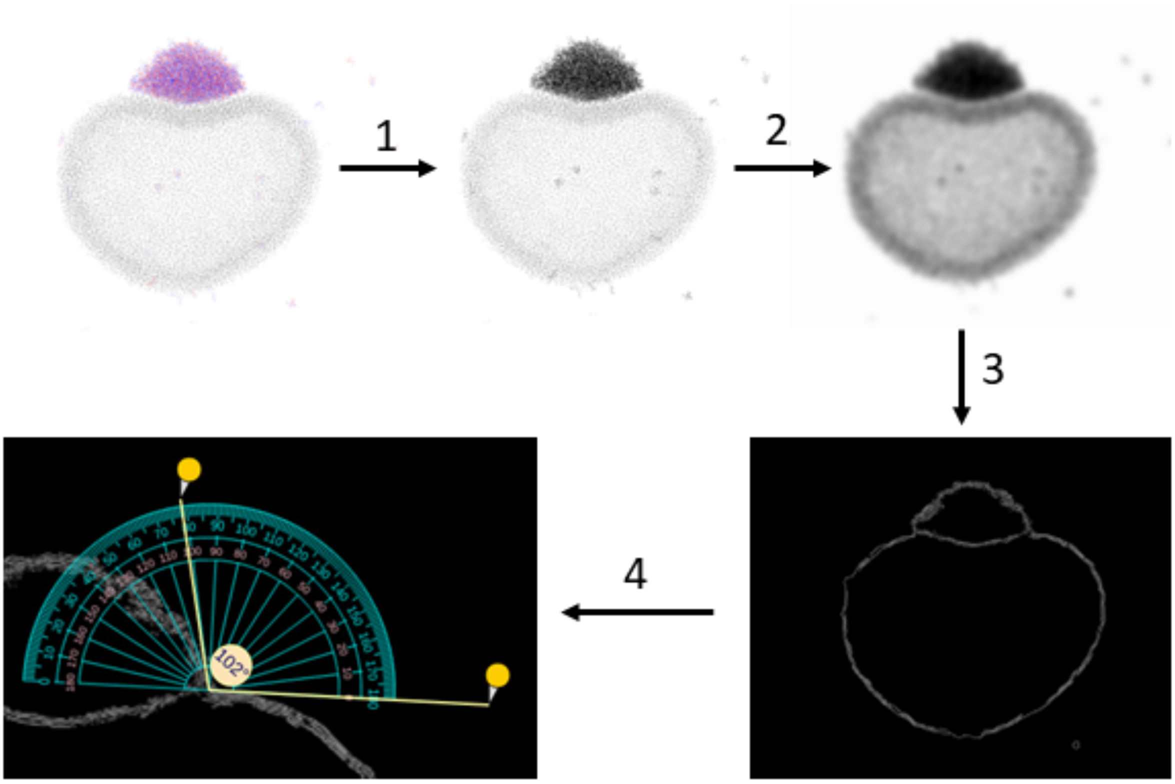

Figure S6. Contact angle determination from simulation snapshots. First, similar to the experimental approach, we find a two-dimensional projection of the system by aligning the center-of-mass (COM) vector connecting the condensate and the vesicle with a box Z-axis, using Visual Molecular Dynamics (VMD) software and convert the colored image into a greyscale one (Step-1). In Step-2, a gaussian blur filter is applied along with gamma correction to produce the input for the edge detection. In Step-3, Canny edge detection algorithm is applied to the output of Step-2. Finally, in Step-4, a web-tool named 'online protractor' (https://www.ginifab.com/feeds/angle_measurement/) is used to measure the intrinsic contact angle. Steps 1 to 3 can be automated, however the main challenge arises in defining smooth and physically meaningful interfaces around both the condensate and the vesicle. Since the simulations are performed at the nanometer scale (unlike experimental systems that operate at the micrometer scale), the extracted boundaries tend to be rugged and noisy. Automated smoothing often introduces significant errors in the measured intrinsic contact angle. Therefore, some degree of manual intervention remains necessary to ensure accuracy.

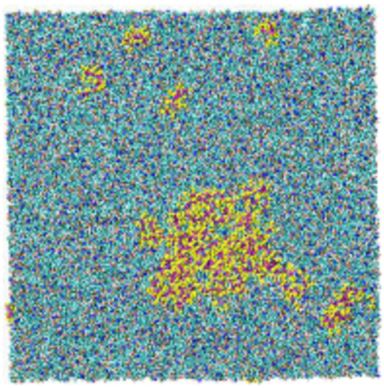

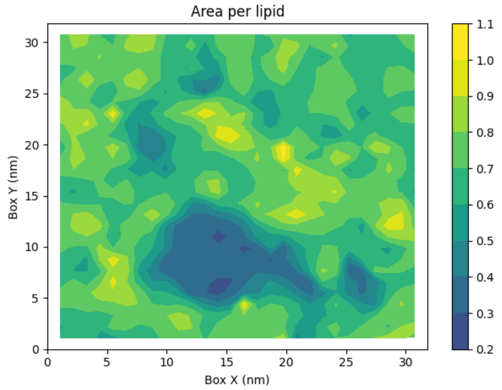

Figure S7. Additional analysis of area per lipid (APL) from MARTINI simulations. (Left) A representative snapshot of the membrane-adsorbed coacervate viewed from the top. (Right) A spatially resolved contour map of the APL in nm^2 unit shows low values of APL near the coacervate-membrane contact region where DOPS lipids are de-mixed from the upper leaflet. This suggests the left shoulder in the APL distribution shown in Figure 5f of the main text arises due to the packed and de-mixed DOPS lipids beneath the coacervate.

Table S2. Average values of the area per lipid (APL) of two different kinds of lipids with and without the coacervate adsorbed on it.

	**Without adsorbed** ***coacervate***		**With adsorbed** ***coacervate***	
	DOPC (nm^2)	DOPS (nm^2)	DOPC (nm^2)	DOPS (nm^2)
Pure DOPC (MARTINI)	0.660 ± 0.003	---	0.645 ± 0.003	---
10% DOPS (MARTINI)	0.658 ± 0.003	0.673 ± 0.005	0.657 ± 0.003	0.572 ± 0.004
20% DOPS (MARTINI)	0.655 ± 0.003	0.674 ± 0.005	0.648 ± 0.003	0.568 ± 0.007

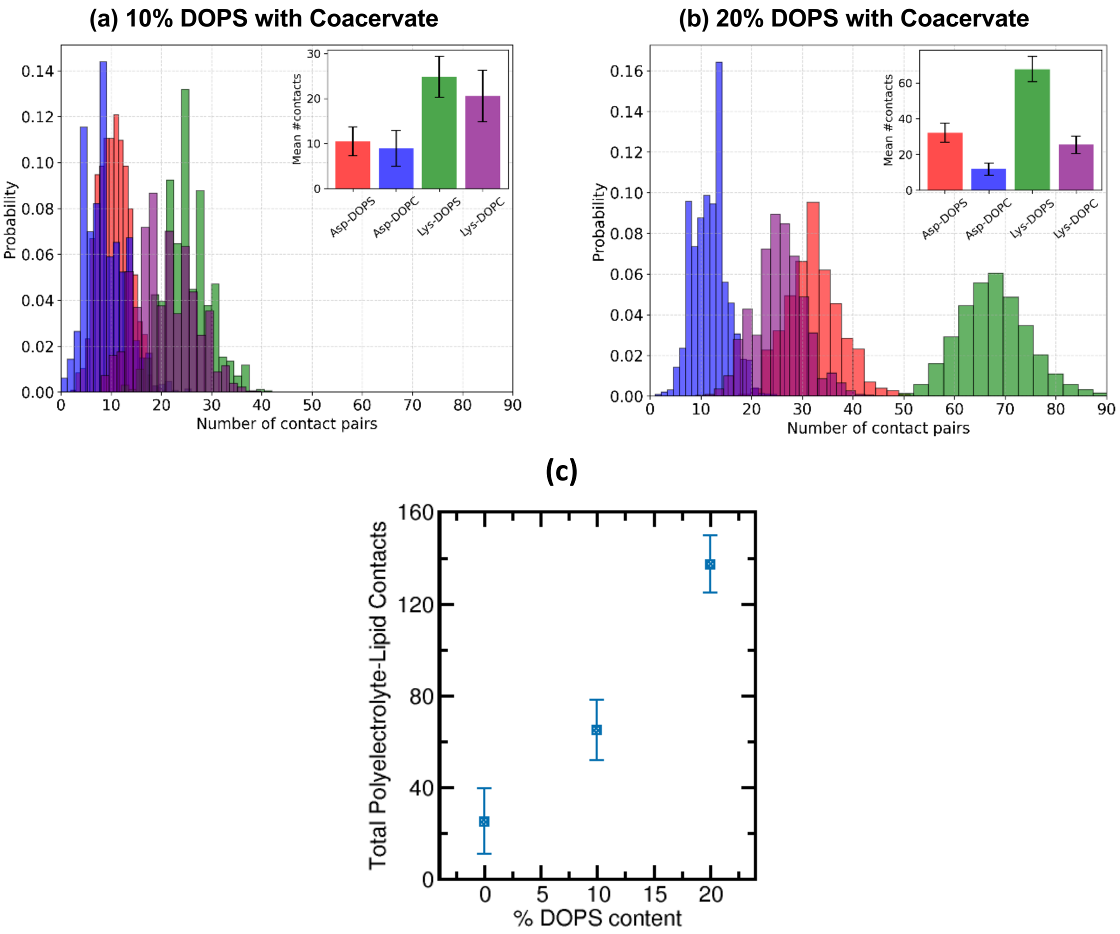

Figure S8. Distribution of the number of contacts between different components in the system. (Inset) Time-averaged number of contacts with error bars for (a) 10% DOPS and (b) 20% DOPS systems. The plots indicate that Lysine residues form more contact with lipids, particularly DOPS, due to the oppositely charged headgroups. Although Aspartate residues typically repel DOPS, their attachment to Lysine residues results in their co-movement, leading to increased contacts with DOPS. The enthalpic loss from the Asp-DOPS interaction is compensated by the favorable and more frequent Lys-DOPS contacts. Additionally, underneath the Asp-Lys coacervate, there is an increased population of DOPS, leading to fewer DOPC contacts overall. (c) Variation of the total number of contacts (r_{cut} = 5Å) between polyelectrolyte backbone beads and lipid heads against the % DOPS content, obtained from the MARTINI simulations.

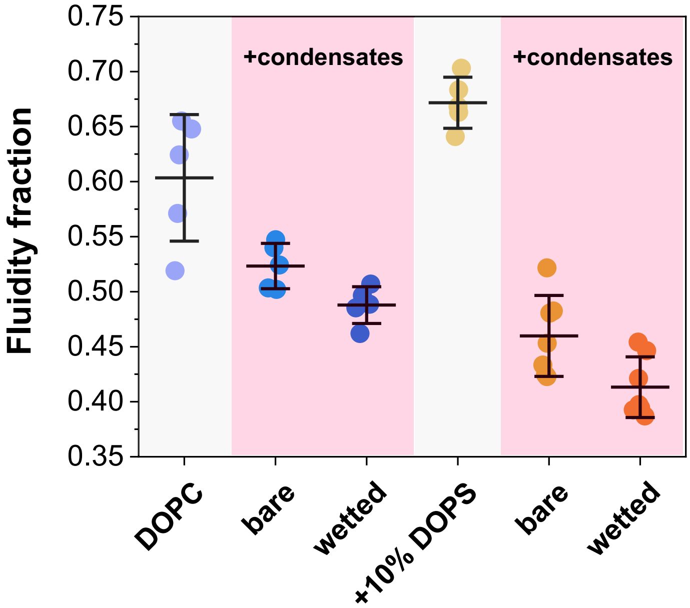

Figure S9. Summary of fluidity fractions for the studied conditions. The membranes in absence of the condensate suspension (i.e. in contact with the same buffer; only the peptides missing) are shown in the gray shaded regions and the vesicles in contact with the condensate suspension in the pink shaded region.

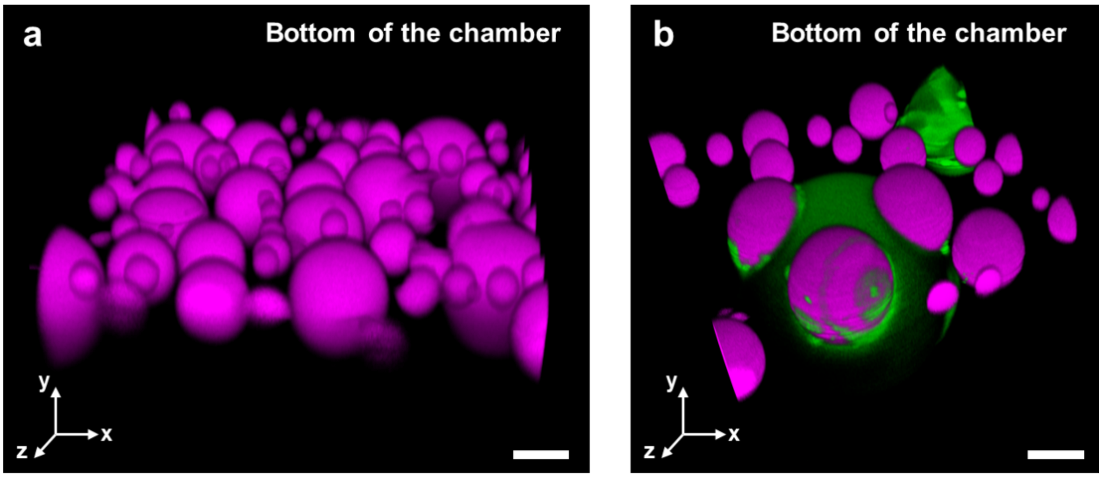

Figure S10. Additional imaging analysis of condensate and GUV. (a) 3D projection of $K_{10}D_{10}$ condensates. Condensates do not wet the glass surface and remain spherical. (b) 3D projection of condensates interacting with a GUV. The condensates wet and reshape at the membrane surface. The GUV does not wet the chamber bottom and gets immobilized by the interaction with the condensates. Scale bars are 5 µm.
